# Prevalence and Ocular Biometric Characteristics of Myopia in Primary Angle Closure Disease in Rural China: The Handan Eye Study

**DOI:** 10.1167/iovs.63.12.19

**Published:** 2022-11-14

**Authors:** Yuanbo Liang, Ruyue Shen, Weihe Zhou, Sujie Fan, Poemen P. Chan, Clement C. Y. Tham, Nathan Congdon, David S. Friedman, Ningli Wang

**Affiliations:** 1 Clinical & Epidemiological Eye Research Center, Eye Hospital of Wenzhou Medical University, Wenzhou, Zhejiang, China; 2 Glaucoma Institute, Wenzhou Medical University, Wenzhou, Zhejiang, China; 3 Department of Ophthalmology and Visual Sciences, The Chinese University of Hong Kong, Hong Kong, China; 4 Handan Eye Hospital, Handan, China; 5 Centre for Public Health, Queen's University, Belfast, United Kingdom; 6 Orbis International, New York, New York, United States; 7 Glaucoma Center of Excellence, Massachusetts Eye and Ear, Harvard Medical School, Boston, Massachusetts, United States; 8 Beijing Tongren Eye Center, Beijing Tongren Hospital, Capital Medical University; Beijing Ophthalmology & Visual Science Key Lab, Beijing, China

**Keywords:** prevalence, angle closure, glaucoma, myopia, axial length

## Abstract

**Purpose:**

The purpose of this study was to determine the prevalence of myopia among patients with primary angle closure disease (PACD) in rural China and their ocular biometric characteristics.

**Methods:**

Study subjects were recruited from the Handan Eye Study. A/B-mode scan (Cine Scan, Quantel Medical, Cedex, France) was used to measure the axial length, anterior chamber depth (ACD), and lens thickness (LT). PACD was defined as the anterior chamber angle being considered closed when 180 degrees or more of the posterior pigmented trabecular meshwork were not visible on the gonioscopy. Myopia was defined as a spherical equivalent (SE) refractive error ≤−0.5 diopter (D). Persons who did not meet PACD definition were classified as the open-angle (OA) group.

**Results:**

The overall prevalence of myopia in persons with PACD was 13.7% (11.6% in primary angle closure suspect [PACS], 21.6% in primary angle closure [PAC], 62.5% in primary angle closure glaucoma [PACG]). The age-specific prevalence of myopia in PACD eyes was 41.7% at 30 to 39 years old, 12.3% at 40 to 49 years old, 8.7% at 50 to 59 years old, 10.7% at 60 to 69 years old, and 31.7% at age 70 years and over. PACD had shorter AL (22.2 ± 0.8 vs. 22.9 ± 0.9 mm, *P* < 0.001), shallower ACD (2.3 ± 0.3 vs. 2.8 ± 0.4 mm, *P* < 0.001), and greater LT (5.0 ± 0.5 vs. 4.7 ± 0.5 mm, *P* < 0.001). PACD had even thicker lenses and deeper ACD with age than those with OA (all *P* ≤ 0.025) from 30 years to 70 years of age and over.

**Conclusions:**

Myopia was common among persons with PACD who were less than 40 years of age in this rural Chinese population, and over half of those with PACG were myopic.

Myopia is the most common disorder affecting distance vision globally. It is estimated that approximately half of the world's population will be myopic by 2050.^1^^–^^3^ Population studies revealed a prevalence of myopia in China as high as 74.2% in adult populations,^4^^–^^6^ and 80% in children and adolescents.^7^^,^^8^ Previous studies suggested that myopia could be “protective” against angle closure glaucoma because of the divergent ocular biometry of these two conditions^9^^,^^10^: the characteristic short axial length, shallow anterior chamber, and thick lens in angle closure glaucoma versus the long axial length and deep anterior chamber in myopic eyes.^11^^–^^15^

Given the distinctive ocular biometrics of the two conditions, the increasing number of people with myopia may affect the prevalence of all forms of primary angle closure disease (PACD), including primary angle closure suspect (PACS), primary angle closure (PAC), and primary angle closure glaucoma (PACG).^16^ A previous hospital-based study reported that patients with myopia with angle closure had long axial length but shallow anterior chamber,^17^ whereas a simulation study showed that the shallow anterior chamber, rather than refractive status, was the major risk factor for angle closure.^18^ To date, there is no population-based study investigating the prevalence and ocular biometric parameters of persons with myopia and angle closure in detail. Such information will be helpful for interpreting the occurrence of myopia in angle closure and studying the influence of the increasing prevalence of myopia on the future prevalence of angle closure.

The purpose of this study is to estimate the age- and gender-specific prevalence of myopia among persons with PACD on a population basis, and to describe ocular biometric characteristics of those with PACD and myopia based on data collected in the Handan Eye Study I (HES I) in 2006 to 2007.

## Methods

### Study Population

The HES was a population-based study designed to estimate the prevalence of major blinding eye diseases in a rural region of northern China. The study design, methodology, and baseline data have been described in detail previously.^19^^,^^20^ In brief, using a stratified, clustered, multistaged sampling method, 7557 subjects aged >30 years were identified in 13 randomly selected villages in Yongnian County, Handan City, Hebei Province, China. All examinations of eligible individuals were conducted from October 2006 to October 2007. In total, 6830 (90.4%) subjects participated in the study, and the data of 6791 participants (99.4%) with complete demographic and clinical information were analyzed.

Ethical approval for this study was obtained from the Ethics Committee of Beijing Tongren Hospital. All participants provided written informed consent, and the tenets of the Declaration of Helsinki were followed throughout.

### Examinations

All participants underwent comprehensive ophthalmic examinations. Best-corrected visual acuity (BCVA) was measured using a logarithm of the minimum angle of resolution (logMAR) chart at a distance of 4 meters. Refraction was carried out with an autorefractor without cycloplegia (KR8800, Topcon, Tokyo, Japan). Axial length (AL), anterior chamber depth (ACD), and lens thickness (LT) were measured with A/B-mode scan ultrasound (Cine Scan, Quantel Medical, Clermont-Ferrand, France). Intraocular pressure (IOP) was measured by a Kowa applanation tonometer (HA-2; Kowa Company Ltd., Tokyo, Japan). The retinal nerve fiber layer (RNFL) was imaged using the Zeiss GDx VCC system (Zeiss Company, Dublin, CA, USA). Slit-lamp biomicroscopy (Topcon SL-2F; Topcon, Tokyo, Japan) was used to assess the limbal anterior chamber depth (LACD) with a modified van Herick grading system.^21^

Gonioscopy was performed on those with LACD less than or equal to 40% of limbal corneal thickness and on 1 in 10 participants with LACD greater than 40% of limbal corneal thickness. Gonioscopy was carried out in four quadrants with a Goldmann lens. A four-mirror Sussman lens (Ocular Instruments, Bellevue, WA, USA) was used if the examination was not satisfactory. The characteristics of the anterior chamber angle were recorded using the Spaeth Gonioscopic Grading System.^7^ Non-mydriatic fundus photographs of the optic nerve head and macula were acquired with a digital fundus camera (Topcon TRC-NW6S/7S; Topcon Corporation, Tokyo, Japan). Digital color stereoscopic photographs (Canon CR-DGi; Canon, Inc., Tokyo, Japan) and tomographic imaging (Heidelberg Retina Tomography HRT II; Heidelberg Engineering, Heidelberg, Germany) of the optic nerve head were obtained separately. All participants with angle closure disease or suspected glaucoma (both defined below) underwent SITA standard visual field testing (Humphrey Visual Field Analyzer 750i; Carl Zeiss, Jena, Germany). Tests were repeated 20 minutes later if the Glaucoma Hemifield Test (GHT) was outside the normal limits or borderline. Visual field testing was repeated if the result was unreliable (i.e. fixation loses >20%, false positives >33%, or false negatives >33%).

Other systemic variables included blood pressure, height, and weight, which were measured by qualified nurses. Body mass index (BMI) was calculated using data on height and weight. A detailed questionnaire was used to record the medical history and systemic diseases, including previous ocular surgery, co-existing diabetes mellitus (DM), and hypertension.

### Definitions

Subjects were categorized into the open-angle (OA) group and the PACD group; the latter included PACS, PAC, and PACG. The classification and definition of PACD were as recommended by the International Society for Geographical and Epidemiological Ophthalmology (ISGEO).^22^ PACS was defined as 180 or more degrees of nonvisible posterior trabecular meshwork on gonioscopy in either eye, without permanent synechial closure and elevated IOP. PAC was defined as PACS with peripheral anterior synechiae (PAS) and/or IOP ≥21 mm Hg without glaucomatous optic neuropathy (GON). PACG was defined as PAC with evidence of GON. Subjects were classified as in the PACD group when one or both eyes had PACD. If one eye was PACG and the contralateral eye was PACS or PAC, that person was classified as in the PACG group; if one eye had PAC and the contralateral eye was PACS, that person was classified as into the PAC group. Subjects who did not meet the definition of PACD were classified into the OA group. Myopia was defined as a spherical equivalent (SE) refractive error ≤−0.5 diopters (D) in either eye. Low, moderate, and high myopia were defined as −2.0 D < SE ≤−0.5 D, −5.0 D < SE ≤−2.0 D, and SE ≤−5.0 D, respectively.^17^

### Statistical Analysis

All statistical analyses were performed using SAS software 9.4 (SAS Institute Inc., Cary, NC, USA). The results were described as mean and standard deviation (mean ± SD) for continuous variables with a normal distribution, and as medians and interquartile (Q1-Q3) otherwise. Categorical variables were described as frequency and percentage. The normality of the variables was examined using the Shapiro-Wilk test. The *t*-test and the analysis of variance (ANOVA) were used to compare continuous variables with normal distribution. The Mann-Whitney *U* test and the Kruskal-Wallis test were used to analyze continuous variables with non-normal distribution among different groups, followed by the post hoc test of Dwass, Steel, Critchlow, Fligner (DSCF).^23^ The chi-square test was used to assess differences in categorical variables. Prevalence was calculated as the ratio of the number of individuals with myopia to the total number of subjects in each subgroup. Only the right eye of persons with OA, the only affected eye in those with unilateral glaucoma, and the more severely affected eye in persons with bilateral PACD were included in the analyses. A *P* value < 0.05 was considered statistically significant. Sensitivity analysis was performed by defining PACS as 270 or more degrees without visible posterior trabecular meshwork on gonioscopy.

## Results

Among a total of 6830 participants in the HES, 39 (0.6%) were excluded due to a history of cataract or refractive surgery, leaving 6791 (99.4%) participants included in the final analysis. Among these, 5855 (86.2%) were subjects with OA, 936 (13.8%) were diagnosed as having PACD, including 795 (11.7%) with PACS, 116 (1.7%) with PAC, and 25 (0.3%) with PACG.

Demographic and clinical characteristics of participants are summarized in Table 1. Compared to subjects with OA, persons with PACD were more likely to be women (75.9% vs. 50.2%), older (59.9 ± 9.3 years vs. 51.1 ± 12.1 years), were more hyperopic SE (0.5 [0.0–1.3] D vs. −0.1 [−0.5 to 0.4] D), had shorter AL (22.2 ± 0.8 mm vs. 22.9 ± 0.9 mm), shallower ACD (2.3 ± 0.3 mm vs. 2.8 ± 0.4 mm), thicker lenses (5.0 ± 0.5 mm vs. 4.7 ± 0.5 mm), worse BCVA (0.8 ± 0.3 vs. 0.9 ± 0.3), thinner RNFL thickness (0.2 ± 0.1 mm vs. 0.3 ± 0.1 mm), and higher prevalence of diabetes (4.6% vs. 2.3%) and hypertension (64.4% vs. 44.9%, all *P* < 0.001). There were no significant differences in BMI or IOP between the two groups. Among the PACD subgroups, the mean age was oldest for those with PACG (64.4 ± 12.5 years), followed by PAC (61.5 ± 9.7 years), and PACS (59.5 ± 9.1 years). Mean IOP ranged from PACS (14.7 ± 2.6 mm Hg) to PAC (18.7 ± 5.1 mm Hg) to PACG (28.6 ± 15.1 mm Hg). The PACG group (−0.8 [−1.3 to 1.0] D) was more myopic than those with PACS (0.5 [0.0-1.3] D) or PAC (0.5 [0.0-1.3] D, *P* = 0.004 and *P* = 0.027, respectively). There were no significant differences in gender, AL, ACD, LT, BMI, and prevalence of DM or hypertension among the three groups.

**Table 1.** Demographic and Clinical Characteristics of Included Participants

				PACD Subgroups			*P* Value^†^		
Variables	OA (*n* = 5855)	PACD (*n* = 936)	*P* Value^*^	PACS (*n* = 795)	PAC (*n* = 116)	PACG (*n* = 25)	PACS vs. PAC	PACS vs. PACG	PAC vs. PACG
Gender (female, %)	50.2	75.9	**<** **0.001**	76.0	76.7	68.0	0.860	0.360	0.360
Age, y	51.1 ± 12.1	59.9 ± 9.3	**<** **0.001**	59.5 ± 9.1	61.5 ± 9.7	64.4 ± 12.5	0.076	**0.013**	0.192
BMI, kg/m^2^	24.5 ± 3.8	24.4 ± 3.7	0.201	24.3 ± 3.7	24.9 ± 3.8	24.2 ± 4.3	0.173	0.998	0.814
IOP, mm Hg	15.0 ± 2.8	15.6 ± 4.6	0.065	14.7 ± 2.6	18.7 ± 5.1	28.6 ± 15.1	**<** **0.001**	**<** **0.001**	**0.044**
SE (D)	−0.1 (−0.5 to 0.4)	0.5 (0.0-1.3)	**<** **0.001**	0.5 (0.0-1.3)	0.5 (0.0-1.3)	−0.8 (−1.3 to 1.0)	0.700	**0.004**	**0.027**
AL, mm	22.9 ± 0.9	22.2 ± 0.8	**<** **0.001**	22.2 ± 0.7	22.1 ± 1.1	22.2 ± 0.5	0.142	0.993	0.686
ACD, mm	2.8 ± 0.4	2.3 ± 0.3	**<** **0.001**	2.3 ± 0.3	2.3 ± 0.3	2.3 ± 0.4	0.879	0.964	0.999
LT, mm	4.7 ± 0.5	5.0 ± 0.5	**<** **0.001**	5.0 ± 0.5	4.9 ± 0.5	4.8 ± 0.4	0.986	0.157	0.211
BCVA	0.9 ± 0.3	0.8 ± 0.3	**<** **0.001**	0.8 ± 0.2	0.7 ± 0.3	0.3 ± 0.3	**0.003**	**<** **0.001**	**<** **0.001**
RNFL thickness, mm	0.3 ± 0.1	0.2 ± 0.1	**<** **0.001**	0.3 ± 0.1	0.3 ± 0.1	0.2 ± 0.1	**0.030**	**<** **0.001**	**<** **0.001**
Diabetic mellitus, %	2.3	4.6	**<** **0.001**	4.8	4.4	0	1.000	0.783	0.864
Hypertension, %	44.9	64.4	**<** **0.001**	62.9	72.4	76.0	0.138	0.543	1.000

PACD, primary angle closure disease; PACS, primary angle closure suspect; PAC, primary angle closure; PACG, primary angle closure glaucoma; OA, open angle; BMI, body mass index; IOP, intraocular pressure; SE, spherical equivalent; AL, axial length; ACD, anterior chamber depth; LT, lens thickness; BCVA, best corrected visual acuity; RNFL, retinal nerve fiber layer.

Boldface values indicate statistical significance. Only the right eye of persons with open angle, the only affected eye in those with unilateral glaucoma, and the more severely affected eye in persons with bilateral glaucoma were included in the analyses.

* *P* value was calculated by the *t*-test for normally distributed continuous variables, Mann-Whitney *U* test for non-normally distributed continuous variables, and chi-square test for categorical variables.

† *P* value was calculated by 1-way ANOVA for normally distributed continuous variables, and Kruskal-Wallis test followed by the post hoc test of Dwass, Steel, Critchlow, Fligner (DSCF) for non-normally distributed variables, and chi-square test for categorical variables.

**Figure 1.**
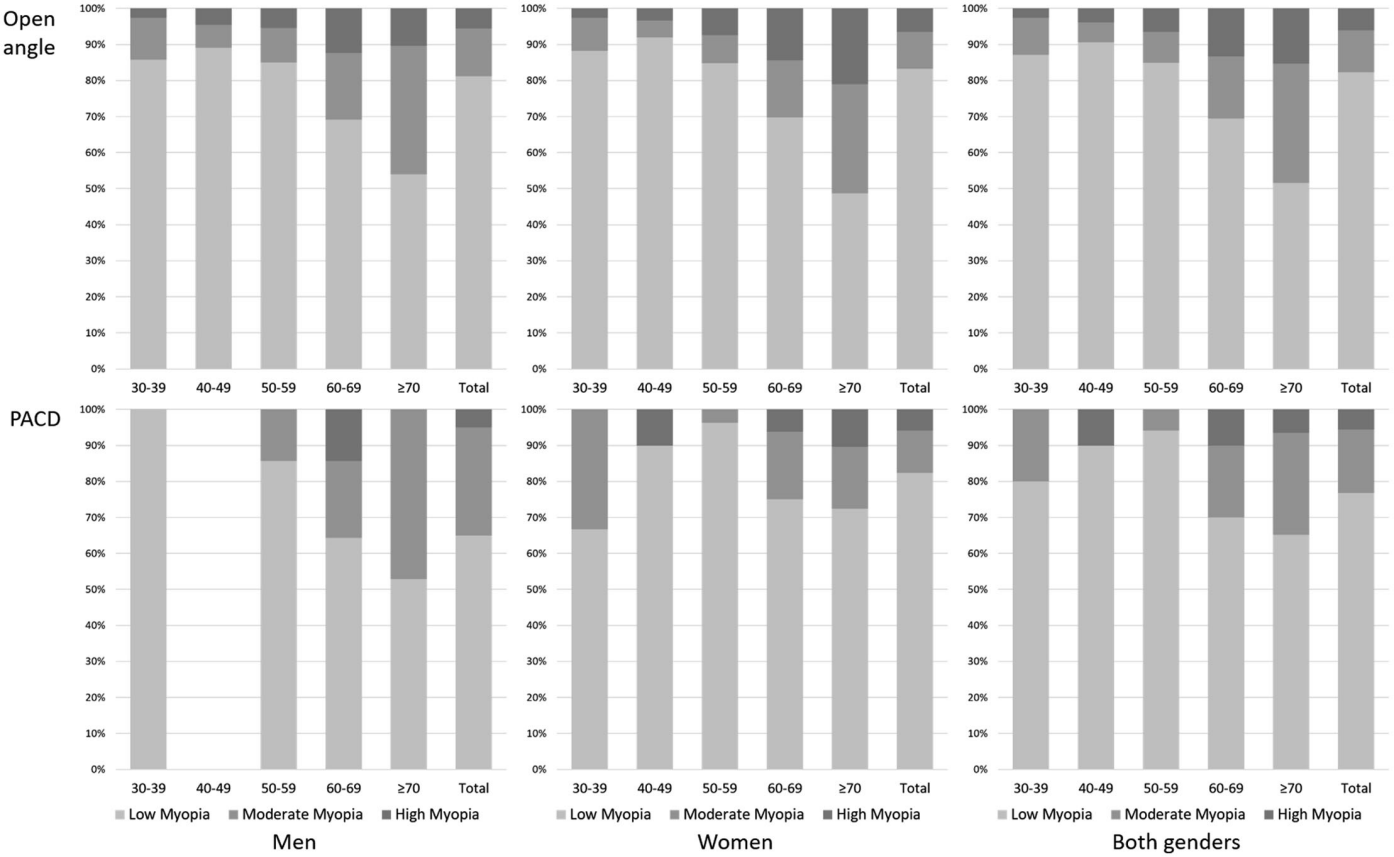
Age- and gender-specific rates of low, moderate, and high myopia in the OA group and the PACD group.

The age- and gender-specific prevalence of myopia in subjects with OA and persons with PACD are summarized in Table 2. A total of 1707 (30.5%) persons had myopia in the OA group, and 125 (13.7%) persons had myopia in the PACD group. The age-specific prevalence of myopia among subjects with OA was 48.5% at 30 to 39 years of age, 31.7% at 40 to 49 years of age, 21.3% at 50 to 59 years of age, 20.2% at 60 to 69 years of age, and 41.4% in those aged 70 years and older. The age-specific prevalence of myopia among persons with PACD was 41.7% at 30 to 39 years of age, 12.3% at 40 to 49 years of age, 8.7% at 50 to 59 years of age, 10.7% at 60 to 69 years of age, and 31.7% in those aged 70 years and older. Among those with PACD, there were 96 (76.8%) persons with low myopia, 22 (17.6%) persons with moderate myopia, and 7 (5.6%) persons with high myopia (Fig. 1). The prevalence of axial myopia (defined as AL >24.0 mm) was 6.6% in the OA group and 0.6% in the PACD group (Supplementary Table S1). OA had a higher proportion of moderate-to-high myopia than PACD (5.4% vs. 3.2%, *P* = 0.005; Supplementary Table S2). The prevalence of nuclear cataract was higher among patients with PACD with myopia (32.8%) compared with those patients with PACD without myopia (6.0%; Supplementary Table S3).

**Table 2.** Age- and Gender-Specific Prevalence of Myopia in Subjects With OA and Persons With PACD in the Handan Eye Study^*^

			OA			PACD	
Group	Age, y	No. at Risk	No.	%	No. at Risk	No.	%
Men							
	30–39	531	267	50.3	2	2	100.0
	40–49	576	174	30.2	11	0	0.0
	50–59	1033	200	19.4	91	7	7.7
	60–69	450	81	18.0	80	14	17.5
	≥70	199	87	43.7	39	17	43.6
	Total	2789	809	29.0	223	40	17.9
*P* for trend				***P* < 0.001**			***P* < 0.001**
Women							
	30–39	648	305	47.1	10	3	30.0
	40–49	639	211	33.0	70	10	14.3
	50–59	989	230	23.3	302	27	8.9
	60–69	329	76	23.1	200	16	8.0
	≥70	195	76	39.0	106	29	27.4
	Total	2800	898	32.1	688	85	12.4
*P* for trend				***P* < 0.001**			***P* = 0.022**
Both genders							
	30–39	1179	572	48.5	12	5	41.7
	40–49	1215	385	31.7	81	10	12.3
	50–59	2022	430	21.3	393	34	8.7
	60–69	779	157	20.2	280	30	10.7
	≥70	394	163	41.4	145	46	31.7
	Total	5589	1707	30.5	911	125	13.7
*P* for trend				***P* < 0.001**			***P* < 0.001**

Prevalence was calculated as the ratio of the number of individuals with myopia to the total number of individuals in each subgroup. Subjects were classified as the PACD group when one eye or both eyes had PACD. Subjects who did not meet the definition of PACD were classified into the open angle group.

OA, open angle; PACD, primary angle closure disease. Boldface values indicate statistical significance.

* Persons with missing values of refractive error (*n* = 266 in the OA group, and 25 in the PACD group) were excluded.

**Figure 2.**
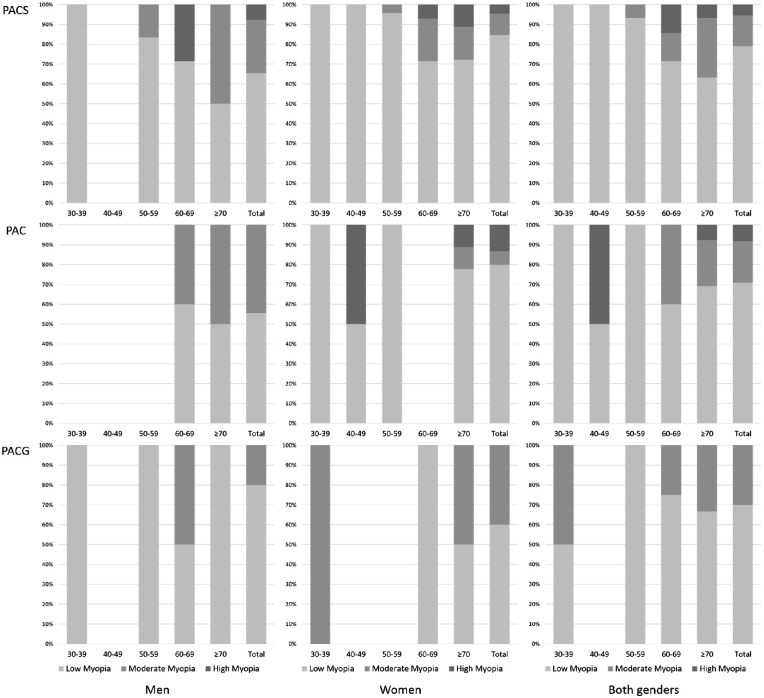
Age- and gender-specific rates of low, moderate, and high myopia in the PACS, PAC, and PACG subgroups.

Table 3 shows the age- and gender-specific prevalence of myopia in different subgroups of PACD. The number of persons with myopia was 91 (11.6%) in the PACS group, 24 (21.6%) in the PAC group, and 10 (62.5%) in the PACG group, respectively. The rates of low, moderate, and high myopia among PACS, PAC, and PACG subgroups are summarized in Figure 2.

**Table 3.** Age- and Gender-Specific Prevalence of Myopia Among PACD Subgroups in the Handan Eye Study^*^

			PACS			PAC			PACG	
Groups	Age, y	No. at Risk	No.	%	No. at Risk	No.	%	No. at Risk	No.	%
Men										
	30–39	1	1	100.0	0	0	0.0	1	1	100.0
	40–49	10	0	0.0	1	0	0.0	0	0	0.0
	50–59	85	6	7.1	4	0	0.0	2	1	50.0
	60–69	64	7	10.9	14	5	35.7	2	2	100.0
	≥70	30	12	40.0	7	4	52.0	2	1	50.0
	Total	190	26	13.7	26	9	34.6	7	5	71.4
*P* for trend				***P* < 0.001**			*P* **=** 0.052			*P* **=** 0.608
Women										
	30–39	7	1	14.3	2	1	50.0	1	1	100.0
	40–49	63	8	12.7	7	2	28.6	0	0	0.0
	50–59	265	24	9.1	35	3	8.6	2	0	0.0
	60–69	173	14	8.1	23	0	0.0	4	2	50.0
	≥70	86	18	20.9	18	9	52.0	2	2	100.0
	Total	594	65	10.9	85	15	17.6	9	5	55.6
*P* for trend				*P* **=** 0.122			*P* **=** 0.113			*P* **=** 0.715
Both genders										
	30–39	8	2	25.0	2	1	50.0	2	2	100.0
	40–49	73	8	11.0	8	2	25.0	0	0	0.0
	50–59	350	30	8.6	39	3	7.7	4	1	25.0
	60–69	237	21	8.9	37	5	13.5	6	4	66.7
	≥70	116	30	25.9	25	13	52.0	4	3	75.0
	Total	784	91	11.6	111	24	21.6	16	10	62.5
*P* for trend				***P* = 0.002**			***P* = 0.010**			*P* **=** 0.924

Prevalence was calculated as the ratio of the number of individuals with myopia to the total number of subjects in each subgroup. The diagnosis is based on the condition of the more severe form of angle closure, i.e., if one eye was PACG and the contralateral eye was PACS or PAC, that person was classified as PACG group; if one eye had PAC and the contralateral eye was PACS, that person was classified as PAC.

PACS, primary angle closure suspect; PAC, primary angle closure; PACG, primary angle closure glaucoma. Boldface values indicate statistical significance.

* Persons with missing values of refractive error (*n* = 25) were excluded.

**Figure 3.**
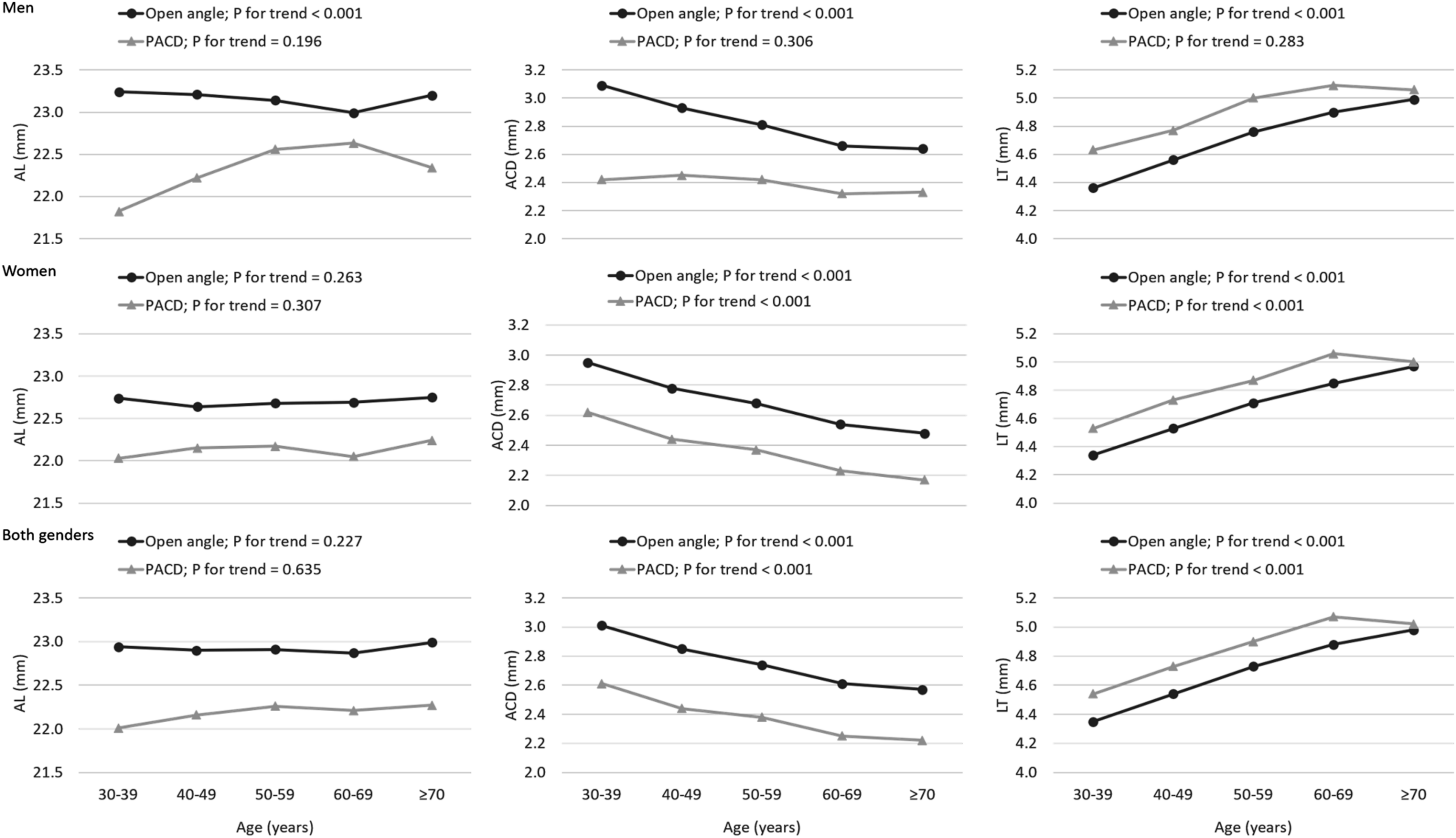
Age- and gender-specific ocular biometric characteristics in the OA group and the PACD group.

Table 4 shows the age- and gender-specific ocular biometric parameters among subjects with OA and PACD. There was no significant change in AL from age 30 years to 70+ years in either the OA group or the PACD group (*P* for trend = 0.227 and 0.635, respectively). Compared to the OA group, PACD eyes had deeper ACD and thicker LT (all *P* ≤ 0.025) in patients from 30 years to 70 years and over. Figure 3 shows the trend of ocular biometric characteristics with age between persons with OA and those with PACD. Our sensitivity analysis showed similar results, which is summarized in Supplementary Tables S4, S5, and S6. Compared with non-myopic PACD persons, myopic PACD persons had shorter AL (22.5 ± 1.1 vs. 22.2 ± 0.7, *P* = 0.004; Supplementary Table S7).

**Table 4.** Age- and Gender-Specific Ocular Biometric Parameters in the Handan Eye Study

					PACD Subgroups		
Variables	Age, y	OA	PACD	*P* Value^*^	PACS	PAC	PACG
Men							
SE (D)	30–39	−0.5 (−0.9 to 0.1)	−0.9 (−1.3 to 0.0)	0.243	–^†^	–^†^	–^†^
	40–49	−0.1 (−0.5 to 0.1)	0.3 (0.0-0.5)	**<** **0.001**	0.3 (0.0-0.5)	–^†^	–^†^
	50–59	0.1 (−0.3 to 0.5)	0.5 (0.1-1.0)	**<** **0.001**	0.5 (0.1-1.0)	0.5 (0.2-1.3)	−0.6 (−1.1 to 0.0)
	60–69	0.4 (−0.3 to 1.0)	0.8 (0.0-1.3)	**0.025**	0.8 (0.2-1.3)	0.2 (−0.6 to 1.4)	−2.6 (−4.8 to 0.0)
	≥70	−0.1 (−1.5 to 0.9)	−0.3 (−1.0 to 0.6)	0.692	−0.1 (−1.2 to 0.8)	−0.5 (−2.0 to 0.4)	−0.6 (−1.0 to 0.0)
*P* for trend		***P* < 0.001**	***P* = 0.002**		***P* = 0.009**	*P* = 0.517	*P* = 0.610
AL, mm	30–39	23.2 ± 0.8	21.8 ± 0.0	0.054	–^†^	–^†^	–^†^
	40–49	23.2 ± 0.9	22.2 ± 0.6	**<** **0.001**	22.2 ± 0.6	–^†^	–^†^
	50–59	23.1 ± 0.8	22.6 ± 0.7	**<** **0.001**	22.6 ± 0.7	21.4 ± 1.0	22.3 ± 1.0
	60–69	23.0 ± 1.0	22.6 ± 0.8	**0.003**	22.7 ± 0.8	22.5 ± 0.7	22.1 ± 0.6
	≥70	23.2 ± 0.9	22.3 ± 0.7	**<** **0.001**	22.3 ± 0.7	22.3 ± 0.9	22.8 ± 0.6
P for trend		***P* < 0.001**	*P* = 0.196		*P* = 0.090	*P* = 0.133	*P* = 0.681
ACD, mm	30–39	3.1 ± 0.4	2.4 ± 0.0	0.092	–^†^	–^†^	–^†^
	40–49	2.9 ± 0.4	2.5 ± 0.3	**<** **0.001**	2.5 ± 0.3	–^†^	–^†^
	50–59	2.8 ± 0.4	2.4 ± 0.3	**<** **0.001**	2.4 ± 0.3	2.4 ± 0.2	3.1 ± 0.5
	60–69	2.7 ± 0.4	2.3 ± 0.3	**<** **0.001**	2.3 ± 0.3	2.2 ± 0.3	2.4 ± 0.3
	≥70	2.6 ± 0.3	2.3 ± 0.3	**<** **0.001**	2.3 ± 0.4	2.3 ± 0.3	2.5 ± 0.2
*P* for trend		***P* < 0.001**	*P* = 0.306		***P* < 0.001**	*P* = 0.550	*P* = 0.255
LT, mm	30–39	4.4 ± 0.6	4.6 ± 0.0	0.426	–^†^	–^†^	–^†^
	40–49	4.6 ± 0.4	4.8 ± 0.3	0.050	4.8 ± 0.3	–^†^	–^†^
	50–59	4.8 ± 0.4	5.0 ± 0.4	**<** **0.001**	5.0 ± 0.4	4.8 ± 0.4	4.4 ± 0.5
	60–69	4.9 ± 0.6	5.1 ± 0.6	**0.001**	5.2 ± 0.4	4.9 ± 1.0	4.9 ± 0.6
	≥70	5.0 ± 0.6	5.1 ± 0.7	0.314	5.1 ± 0.7	4.9 ± 0.5	5.0 ± 0.2
*P* for trend		***P* < 0.001**	*P* = 0.283		*P* = 0.067	*P* = 0.961	*P* = 0.650
Women							
SE, D	30–39	−0.4 (−0.8 to 0.0)	−0.2 (−0.8 to 0.3)	0.230	0.1 (−0.3 to 0.5)	−0.4 (−0.5 to 0.0)	–^†^
	40–49	−0.3 (−0.5 to 0.1)	0.0 (−0.3 to 0.5)	**<** **0.001**	0.0 (−0.3 to 0.5)	−0.3 (−0.5 to 0.3)	–^†^
	50–59	0.0 (−0.4 to 0.5)	0.4 (0.0-1.0)	**<** **0.001**	0.4 (0.0-0.9)	0.8 (−0.3 to 1.4)	1.3 (1.3-1.3)
	60–69	0.5 (−0.4 to 1.1)	1.0 (0.5-1.6)	**<** **0.001**	1.0 (0.5-1.7)	1.1 (0.6-1.8)	1.3 (1.3-4.0)
	≥70	0.1 (−1.1 to 1.5)	0.8 (−0.6 to 1.8)	**0.039**	0.9 (−0.3 to 1.8)	−0.3 (−1.7 to 1.9)	1.3 (1.3-2.0)
*P* for trend		***P* < 0.001**	***P* < 0.001**		***P* <0.001**	***P* = 0.019**	*P* = 0.065
AL, mm	30–39	22.7 ± 0.8	22.0 ± 0.7	**0.002**	22.0 ± 0.8	22.0 ± 0.5	22.3 ± 0.0
	40–49	22.6 ± 0.7	22.2 ± 1.1	**<** **0.001**	22.0 ± 0.6	23.1 ± 3.1	–^†^
	50–59	22.7 ± 0.8	22.2 ± 0.7	**<** **0.001**	22.2 ± 0.7	22.1 ± 0.8	22.4 ± 0.5
	60–69	22.7 ± 1.0	22.1 ± 0.7	**<** **0.001**	22.1 ± 0.6	21.9 ± 0.8	22.0 ± 0.6
	≥70	22.8 ± 1.0	22.2 ± 0.6	**<** **0.001**	22.3 ± 0.7	22.0 ± 0.6	22.4 ± 0.4
*P* for trend		*P* = 0.263	*P* = 0.307		*P* = 0.139	*P* = 0.208	*P* = 0.551
ACD, mm	30–39	3.0 ± 0.4	2.6 ± 0.2	**0.001**	2.6 ± 0.2	2.9 ± 0.3	2.5 ± 0.0
	40–49	2.8 ± 0.4	2.4 ± 0.3	**<** **0.001**	2.5 ± 0.3	2.4 ± 0.2	–^†^
	50–59	2.7 ± 0.4	2.4 ± 0.3	**<** **0.001**	2.4 ± 0.3	2.4 ± 0.4	2.3 ± 0.3
	60–69	2.5 ± 0.4	2.2 ± 0.3	**<** **0.001**	2.2 ± 0.3	2.3 ± 0.3	2.2 ± 0.3
	≥70	2.5 ± 0.4	2.2 ± 0.3	**<** **0.001**	2.2 ± 0.3	2.1 ± 0.2	2.0 ± 0.3
*P* for trend		***P* < 0.001**	***P* < 0.001**		***P* < 0.001**	***P* = 0.004**	*P* = 0.241
LT, mm	30–39	4.3 ± 0.5	4.5 ± 0.3	**0.038**	4.6 ± 0.3	4.3 ± 0.1	4.9 ± 0.0
	40–49	4.5 ± 0.5	4.7 ± 0.3	**<** **0.001**	4.7 ± 0.4	4.9 ± 0.3	–^†^
	50–59	4.7 ± 0.4	4.9 ± 0.4	**<** **0.001**	4.9 ± 0.4	4.9 ± 0.5	4.9 ± 0.3
	60–69	4.9 ± 0.5	5.1 ± 0.4	**<** **0.001**	5.1 ± 0.4	5.0 ± 0.4	5.0 ± 0.5
	≥70	5.0 ± 0.6	5.0 ± 0.8	0.144	5.0 ± 0.9	5.2 ± 0.2	4.6 ± 0.4
*P* for trend		***P* < 0.001**	***P* < 0.001**		***P* < 0.001**	***P* = 0.014**	*P* = 0.575
Both genders							
SE, D	30–39	−0.4 (−0.8 to 0.1)	−0.3 (−1.1 to 0.2)	0.505	0.0 (−0.5 to 0.4)	−0.4 (−0.5 to 0.0)	−2.0 (−2.8 to 0.0)
	40–49	−0.3 (−0.5 to 0.1)	0.1 (−0.3 to 0.5)	**<** **0.001**	0.3 (−0.3 to 0.5)	−0.3 (−0.4 to 0.6)	–^†^
	50–59	0.1 (−0.4 to 0.5)	0.5 (0.0-1.0)	**<** **0.001**	0.5 (0.0-0.9)	0.5 (−0.3 to 1.4)	1.3 (−0.5 to 2.1)
	60–69	0.5 (−0.3 to 1.1)	1.0 (0.4-1.5)	**<** **0.001**	1.0 (0.4-1.5)	1.0 (0.2-1.5)	−0.5 (−1.9 to 0.5)
	≥70	0.0 (−1.4 to 1.1)	0.4 (−0.9 to 1.4)	**0.013**	0.6 (−0.5 to 1.5)	−0.5 (−1.7 to 1.1)	−1.2 (−3.6 to 0.4)
*P* for trend		***P* < 0.001**	***P* < 0.001**		***P* < 0.001**	***P* = 0.017**	*P* = 0.191
AL, mm	30–39	22.9 ± 0.8	22.0 ± 0.7	**<** **0.001**	22.0 ± 0.8	22.0 ± 0.5	22.1 ± 0.4
	40–49	22.9 ± 0.9	22.2 ± 1.1	**<** **0.001**	22.1 ± 0.6	23.1 ± 3.1	–^†^
	50–59	22.9 ± 0.8	22.3 ± 0.7	**<** **0.001**	22.3 ± 0.7	22.1 ± 0.8	22.4 ± 0.6
	60–69	22.9 ± 1.0	22.2 ± 0.8	**<** **0.001**	22.2 ± 0.7	22.1 ± 0.9	22.0 ± 0.5
	≥70	23.0 ± 1.0	22.3 ± 0.7	**<** **0.001**	22.3 ± 0.7	22.1 ± 0.7	22.5 ± 0.5

**Table 4.** Continued

					PACD Subgroups		
Variables	Age, y	OA	PACD	*P* Value^*^	PACS	PAC	PACG
*P* for trend		*P* = 0.227	*P* = 0.635		*P* = 0.157	*P* = 0.238	*P* = 0.317
ACD (mm)	30–39	3.0 ± 0.4	2.6 ± 0.2	**<** **0.001**	2.6 ± 0.2	2.9 ± 0.3	2.5 ± 0.1
	40–49	2.9 ± 0.4	2.4 ± 0.3	**<** **0.001**	2.5 ± 0.3	2.4 ± 0.2	–^†^
	50–59	2.7 ± 0.4	2.4 ± 0.3	**<** **0.001**	2.4 ± 0.3	2.4 ± 0.3	2.6 ± 0.5
	60–69	2.6 ± 0.4	2.3 ± 0.3	**<** **0.001**	2.3 ± 0.3	2.3 ± 0.3	2.3 ± 0.3
	≥70	2.6 ± 0.4	2.2 ± 0.3	**<** **0.001**	2.2 ± 0.3	2.2 ± 0.3	2.1 ± 0.4
*P* for trend		***P* < 0.001**	***P* < 0.001**		***P* < 0.001**	***P* = 0.003**	*P* = 0.125
LT, mm	30–39	4.4 ± 0.5	4.5 ± 0.3	**0.025**	4.6 ± 0.3	4.3 ± 0.1	4.8 ± 0.2
	40–49	4.5 ± 0.4	4.7 ± 0.3	**<** **0.001**	4.7 ± 0.3	4.9 ± 0.3	–^†^
	50–59	4.7 ± 0.4	4.9 ± 0.4	**<** **0.001**	4.9 ± 0.4	4.9 ± 0.5	4.7 ± 0.4
	60–69	4.9 ± 0.5	5.1 ± 0.5	**<** **0.001**	5.1 ± 0.4	5.0 ± 0.6	5.0 ± 0.5
	≥70	4.9 ± 0.6	5.0 ± 0.8	**<** **0.001**	5.0 ± 0.9	5.1 ± 0.3	4.7 ± 0.4
*P* for trend		***P* < 0.001**	***P* < 0.001**		***P* <0.001**	*P* = 0.148	*P* = 0.622

PACD, primary angle closure disease; PACS, primary angle closure suspect; PAC, primary angle closure; PACG, primary angle closure glaucoma; OA, open angle; SE, spherical equivalent; AL, axial length; ACD, anterior chamber depth; LT, lens thickness.

Only the right eye of persons with open angle, the only affected eye in those with unilateral glaucoma, and the more severely-affected eye in persons with bilateral glaucoma were included in analyses.

* *P* value was calculated by the *t*-test for normally distributed variables (i.e. AL, ACD, and LT) and Mann-Whitney *U* test for non-normally distributed variables (i.e. SE).

† Not available due to limited sample size. Boldface values indicate statistical significance.

## Discussion

The prevalence of myopia was over 10% in this population with PACD, affecting the majority of participants with PACG. Consistent with other population-based studies, we found that patients with PACD were older, more likely to be women and hyperopic, had shorter AL, shallower ACD, and greater LT.^24^^,^^25^ Although hyperopia is traditionally considered to be a risk factor for PACD because of the characteristic shallow anterior chamber,^9^^,^^26^ the possibility of encountering PACD among persons with myopia should not be ignored. Our findings were in concordance with a previous study, which reported that persons with myopia with angle closure had longer axial length and vitreous length, but similar ACD to those without myopia but with angle closure.^17^ Loh et al.^27^ found that relative lens position is more forward in patients with myopic angle closure. In addition, we observed an age-related tendency toward shallower anterior chambers and thicker lenses in persons with myopia with PACD, consistent with a prior prospective observational study conducted in healthy subjects.^28^ Notably, no significant difference between different age groups was observed in axial length in either the OA or the PACD group. This finding implies that axial length is not likely the cause of myopia in angle closure. Our findings indicate that age-related lens thickening induces a refractive change toward myopia resulting in myopia among many persons with PACD. Our results showing that the prevalence of nuclear cataract is markedly higher in patients with myopic PACD compared with patients with non-myopic PACD also support that the observed myopia among PACD is secondary to nuclear sclerosis. However, this does not explain that the highest myopia prevalence in our PACD group occurred among persons aged 30 to 39 years, whose prevalence was higher than among persons 70+. It is likely that this, and the declining myopia prevalence with age until 70 years, is due to cohort effects, with elevated prevalence among younger persons exposed to more intensive education in recent years.^3^ Another possible explanation, because most cases of myopia were low in magnitude, is that non-cycloplegic autorefraction resulted in greater accommodation in the youngest participants.

The prevalence of myopia among persons with angle closure in our population is lower than reported by a cross-sectional study from Singapore.^17^ They found myopia in 94 (22%) patients of those with angle closure in a hospital-based population.^17^ A likely explanation for the lower prevalence in the current study is the higher overall burden of myopia in Singapore (38.9%),^29^ compared to this rural Chinese population (26.7%).^4^ We also found that the prevalence of myopia in participants with PACS (11.6%) was lower than that in PAC (21.6%) or PACG (62.5%). This may be explained by age differences, with myopia-inducing cataracts being more common (and also playing a pathogenetic role) in older persons with PACG compared to the younger PACS and PAC groups.

We found an increased tendency toward hyperopia with age among participants 30 to 69 years, followed by greater myopia prevalence in those over the age of 70 years. This is likely due at least in part to cohort effects, with younger persons having been exposed to the myogenic impact of the more intensive educational system which has developed in China.^6^^,^^19^^,^^39^ The finding of greater myopia in younger persons is also consistent with population-based studies outside China,^30^^–^^37^ and, in addition to cohort effects,^34^ may also relate to the loss of accommodative ability with increased age, when cycloplegia, which would unmask hyperopia in younger participants, was not used. The myopic shift in those over 70 years of age is likely due to lens opacity, as reported by previous studies.^30^^,^^38^

This study has implications for future ocular epidemiology in China. A simulation study from the Liwan Eye Study found that with the increasing prevalence of myopia, the prevalence of narrow angles decreased only slightly.^18^ At a 10% prevalence of myopia, narrow angles were present in 11.1% of the population, whereas as the prevalence of myopia increased to 60%, the rate of narrow angles remained similar at 9.6%. However, the prevalence of myopia and angle closure varies greatly between different age groups and regions. The Beijing Myopia Progression and Handan Offspring Eye Studies reported the biometric characteristics in a young population with an increasing prevalence of axial myopia.^5^^,^^39^ In rural^5^ and urban^39^ China, the mean SE increased by 1.0 D and 1.93 D, respectively, in a younger cohort, whereas the mean axial length of children at the age of 18 years increased by 0.57 mm in the rural region, and 1.5 mm in the urban area. ACD increased by 0.59 mm and 0.77 mm, respectively. This implies that there is a different pattern of myopia progression between urban and rural regions. This different pattern of myopia progression would have a different impact on the prevalence of angle closure. The current study found axial length had little change with age, whereas ACD was deeper in the younger population. Based on these data, it may be expected that as the prevalence of myopia increases in younger cohorts, the prevalence of angle closure will decrease in our setting. On the other hand, our findings indicate that we should not ignore performing gonioscopy to identify potential angle closure suspect among patients with myopia in the clinical practice.

The main strengths of the current study are its large size and population-based design. We evaluated the prevalence of myopia in angle closure subgroups and compared the refractive status and biometric parameters of such persons on a population basis. There are limitations in our study as well. First, not all participants underwent gonioscopy. We conducted gonioscopy on all participants with LACD of 40% or less and 10% of participants with LACD greater than 40%. In fact, among those persons with LACD >40% who had been performed gonioscopy, we found 58 (0.8%) had PACS, 14 (0.2%) had PAC, and none had PACG. Thus, this strategy of gonioscopy examination did miss some cases with angle closure and might underestimate the prevalence of PAC or PACS in this setting. Second, non-cycloplegic refraction was used in this study, and thus the effects of accommodation on refractive error were not evaluated. The likely impact of this has been discussed in detail above. Third, this study was performed in rural Chinese, so our results may only be applied with caution to urban settings or other ethnic groups. Additionally, other factors, such as the history of diabetes, which may potentially impact the ocular biometric characteristics, were not fully evaluated. Further studies are essential to validate our study findings by considering these confounding factors.

Despite its limitations, this study provides novel population-based data confirming a high prevalence of myopia in persons with angle-closure disease in rural China, and provides unique information about future epidemiologic trends in this population.

## Supplementary Material

Supplement 1
