# Supplementary material for: Prevalence and Ocular Biometric Characteristics of Myopia in Primary Angle Closure Disease in Rural China: The Handan Eye Study
Source: Invest Ophthalmol Vis Sci. 2022 Nov 14;63(12):19. doi: 10.1167/iovs.63.12.19 (PMC9669803; doi:10.1167/iovs.63.12.19)
Supplement: Supplement 1 [file iovs-63-12-19_s001.pdf]

Supplementary Table S1. Age- and gender-specific prevalence of axial myopia in OA and PACD groups.*

		OA			PACD			
**Group**	**Age (years)**	**No. at risk**	**No.**	**%**	**No. at risk**	**No.**	**%**	**P value**
Axial myopia defined as AL>24.0mm								
Men	30-39	370	55	14.9	1	0	0.0	0.676
	40-49	473	56	11.8	10	0	0.0	0.247
	50-59	859	74	8.6	84	0	0.0	**0.005**
	60-69	373	34	9.1	74	1	1.4	**0.023**
	≥70	143	13	9.1	35	1	2.9	0.219
	Total	2218	232	10.5	204	2	1.0	**<0.001**
P for trend				**0.003**			0.148	
Women								
	30-39	530	20	3.8	11	0	0.0	0.511
	40-49	546	11	2.0	67	1	1.5	0.771
	50-59	876	22	2.5	291	2	0.7	0.057
	60-69	273	9	3.3	195	0	0.0	**0.010**
	≥70	121	6	5.0	85	0	0.0	**0.037**
	Total	2346	68	2.9	649	3	0.5	**<0.001**
P for trend				0.858			0.145	
Both genders								
	30-39	900	75	8.3	12	0	0.0	0.297
	40-49	1019	67	6.6	77	1	1.3	0.064
	50-59	1735	96	5.5	375	2	0.5	**<0.001**
	60-69	646	43	6.7	269	1	0.4	**<0.001**
	≥70	264	19	7.2	120	1	0.8	**0.009**
	Total	4564	300	6.6	853	5	0.6	**<0.001**
P for trend				0.137			0.844	
Axial myopia defined as AL>23.3mm								
Men	30-39	370	156	42.2	1	0	0.0	0.394
	40-49	473	214	45.2	10	1	10.0	**0.026**
	50-59	859	340	39.6	84	11	13.1	**<0.001**
	60-69	373	115	30.8	74	10	13.5	**0.002**
	≥70	143	60	42.0	35	1	2.9	**<0.001**
	Total	2218	885	39.9	204	23	11.3	**<0.001**
P for trend				**0.007**			0.305	
Women								
	30-39	530	100	18.9	11	1	9.1	0.410
	40-49	546	78	14.3	67	3	4.5	**0.025**
	50-59	876	125	14.3	291	15	5.2	**<0.001**
	60-69	273	50	18.3	195	4	2.1	**<0.001**
	≥70	121	25	20.7	85	4	4.7	**0.001**
	Total	2346	378	16.1	649	27	4.2	**<0.001**

P for trend				0.911			0.329	
Both genders								
	30-39	900	256	28.4	12	1	8.3	0.124
	40-49	1019	292	28.7	77	4	5.2	**<0.001**
	50-59	1735	465	26.8	375	26	6.9	**<0.001**
	60-69	646	165	25.5	269	14	5.2	**<0.001**
	≥70	264	85	32.2	120	5	4.2	**<0.001**
	Total	4564	1263	27.7	853	50	5.9	**<0.001**
P for trend				0.684			0.334	

Prevalence was calculated as the ratio of the number of individuals with axial myopia to the total number of individuals in each subgroup. Subjects were classified as PACD group when one eye or both eyes had PACD. Subjects who did not meet the definition of PACD were classified into open angle group. OA, open angle; PACD, primary angle closure disease. Boldface values indicate statistical significance. * Persons with missing values of refractive error were excluded.

Supplementary Table S2. The proportion of moderate to high myopia in OA and PACD.

		OA			PACD			
**Group**	**Age (years)**	**No. at risk**	**No.**	**%**	**No. at risk**	**No.**	**%**	**P value**
Men								
	30-39	267	38	14.2	2	0	0.0	0.565
	40-49	174	19	10.9	0	0	0.0	-
	50-59	200	30	15.0	7	1	14.3	0.958
	60-69	81	25	30.9	14	5	35.7	0.718
	≥70	87	40	46.0	17	8	47.1	0.935
	Total	809	152	18.8	40	14	35.0	**0.012**
Women								
	30-39	305	36	11.8	3	1	33.3	0.254
	40-49	211	17	8.1	10	1	10.0	0.826
	50-59	230	35	15.2	27	1	3.7	0.103
	60-69	76	23	30.3	16	4	25.0	0.674
	≥70	76	39	51.3	29	8	27.6	**0.029**
	Total	898	150	16.7	85	15	17.6	0.824
Both genders								
	30-39	1179	74	6.3	12	1	8.3	0.770
	40-49	1215	36	3.0	81	1	1.2	0.366
	50-59	2022	65	3.2	393	2	0.5	**0.003**
	60-69	779	48	6.2	280	9	3.2	0.061
	≥70	394	79	20.1	145	16	11.0	**0.015**
	Total	5589	302	5.4	911	29	3.2	**0.005**

Prevalence was calculated as the ratio of the number of individuals with axial myopia to the total number of individuals in each subgroup. Subjects were classified as PACD group when one eye or both eyes had PACD. Subjects who did not meet the definition of PACD were classified into open angle group. OA, open angle; PACD, primary angle closure disease. Boldface values indicate statistical significance. * Persons with missing values of refractive error were excluded.

Supplementary Table S3. Age- and gender-specific prevalence of nuclear cataract among PACD patients with myopia or without myopia. *

Group	Age (years)	PACD without myopia			PACD with myopia			
		No. at risk	No.	%	No. at risk	No.	%	P value
Men								
	30-39	0	0	0.0	2	0	0.0	-
	40-49	11	0	0.0	0	0	0.0	-
	50-59	84	0	0.0	7	0	0.0	-
	60-69	66	4	6.1	14	6	42.9	**<0.001**
	≥70	22	3	13.6	17	9	52.9	**0.008**
	Total	183	7	3.8	40	15	37.5	**<0.001**
Women								
	30-39	7	0	0.0	3	0	0.0	-
	40-49	60	0	0.0	10	1	10.0	**0.014**
	50-59	275	0	0.0	27	1	3.7	**0.001**
	60-69	184	15	8.2	16	8	50.0	**<0.001**
	≥70	77	25	32.5	29	16	55.2	**0.032**
	Total	603	40	6.6	85	26	30.6	**<0.001**
Both genders								
	30-39	7	0	0.0	5	0	0.0	-
	40-49	71	0	0.0	10	1	10.0	**0.007**
	50-59	359	0	0.0	34	1	2.9	**0.001**
	60-69	250	19	7.6	30	14	46.7	**<0.001**
	≥70	99	28	28.3	46	25	54.3	**0.002**
	Total	786	47	6.0	125	41	32.8	**<0.001**

PACD, primary angle closure disease. Boldface values indicate statistical significance. * Prevalence was calculated as the ratio of the number of individuals with nuclear cataract to the total number of individuals in each subgroup. Nuclear cataract was defined as nuclear opalescence (NO) or nuclear color (NC) ≥ 4. Persons with missing values of Lens Opacities Classification System (LOCS) data were excluded.

Supplementary Table S4. Age- and gender-specific prevalence of myopia in OA subjects and PACD persons in Handan Eye Study.*

			OA			PACD	
**Group**	**Age (years)**	**No. at risk**	**No.**	**%**	**No. at risk**	**No.**	**%**
Men							
	30-39	532	268	50.4	1	1	100.0
	40-49	580	175	30.2	7	0	0.0
	50-59	1079	204	18.9	45	2	4.4
	60-69	477	84	17.6	53	10	18.9
	≥70	218	97	44.5	20	6	30.0
	Total	2886	828	28.7	126	19	15.1
P for trend				**P < 0.001**			**P = 0.032**
Women							
	30-39	655	306	46.7	3	2	66.7
	40-49	680	217	31.9	29	3	10.3
	50-59	1113	244	21.9	177	15	8.5
	60-69	396	81	20.5	133	10	7.5
	≥70	227	82	36.1	75	22	29.3
	Total	3071	930	30.3	417	52	12.5
P for trend				**P < 0.001**			**P = 0.008**
Both genders							
	30-39	1187	574	48.4	4	3	75.0
	40-49	1260	392	31.1	36	3	8.3
	50-59	2192	448	20.4	222	17	7.7
	60-69	873	165	18.9	186	20	10.8
	≥70	445	179	40.2	95	28	29.5
	Total	5957	1758	29.5	543	71	13.1
P for trend				**P < 0.001**			**P < 0.001**

PACS was defined as 270 or more degrees without visible posterior trabecular meshwork on gonioscopy. Prevalence was calculated as the ratio of the number of individuals with myopia to the total number of individuals in each subgroup. Subjects were classified as PACD group when one eye or both eyes had PACD. Subjects who did not meet the definition of PACD were classified into open angle group. OA, open angle; PACD, primary angle closure disease. Boldface values indicate statistical significance. * Persons with missing values of refractive error were excluded.

Supplementary Table S5. Age- and gender-specific prevalence of myopia in OA subjects and PACD persons in Handan Eye Study.*

		PACS			PAC			PACG		
**Group**	**Age (years)**	**No. at risk**	**No.**	**%**	**No. at risk**	**No.**	**%**	**No. at risk**	**No.**	**%**
Men										
	30-39	0	0	0.0%	0	0	0.0	1	1	100.0
	40-49	6	0	0.0%	1	0	0.0	0	0	0.0
	50-59	39	1	2.6%	4	0	0.0	2	1	50.0
	60-69	37	3	8.1%	14	5	35.7	2	2	100.0
	≥70	14	2	14.3%	4	3	75.0	2	1	50.0
	Total	96	6	6.3%	23	8	34.8	7	5	71.4
P for trend				P = 0.096			P = 0.053			P = 0.589
Women										
	30-39	0	0	0.0%	2	1	50.0	1	1	100.0
	40-49	23	1	4.3%	6	2	33.3	0	0	0.0
	50-59	155	13	8.4%	20	2	10.0	2	0	0.0
	60-69	109	9	8.3%	21	0	0.0	3	1	33.3
	≥70	57	11	19.3%	16	9	56.3	2	2	100.0
	Total	344	34	9.9%	65	14	21.5	8	4	50.0
P for trend				**P = 0.030**			P = 0.176			P = 0.773
Both genders										
	30-39	0	0	0.0%	2	1	50.0	2	2	100.0
	40-49	29	1	3.4%	7	2	28.6	0	0	0.0
	50-59	194	14	7.2%	24	2	8.3	4	1	25.0
	60-69	146	12	8.2%	35	5	14.3	5	3	60.0
	≥70	71	13	18.3%	20	12	60.0	4	3	75.0
	Total	440	40	9.1%	88	22	25.0	15	9	60.0
P for trend				**P = 0.008**			**P = 0.027**			P = 0.867

PACS was defined as 270 or more degrees without visible posterior trabecular meshwork on gonioscopy. Prevalence was calculated as the ratio of the number of individuals with myopia to the total number of subjects in each subgroup. The diagnosis is based on the condition of the more severe form of angle closure, i.e., if one eye was PACG and the contralateral eye was PACS or PAC, that person was classified as PACG group; if one eye had PAC and the contralateral eye was PACS, that person was classified as PAC. PACS, primary angle closure suspect; PAC, primary angle closure; PACG, primary angle closure glaucoma. Boldface values indicate statistical significance. * Persons with missing values of refractive error were excluded.

Supplementary Table S6. Age- and gender-specific ocular biometric parameters in Handan Eye Study.

Variables	Age (years)	OA	PACD	P value*	PACD Subgroups		
					PACS	PAC	PACG
Men							
SE (D)	30-39	-0.5 (-0.9--0.1)	-1.3 (-1.3--1.3)	0.670	-†	-†	-†
	40-49	-0.1 (-0.5-0.1)	0.4 (0.3-1.0)	0.083	0.3 (0.2-0.9)	1.0 (1.0-1.0)	-†
	50-59	0.1 (-0.3-0.5)	0.8 (0.5-1.4)	<0.001	0.8 (0.5-1.3)	0.5 (0.2-1.3)	0.6 (-1.1-0.0)
	60-69	0.4 (-0.3-1.0)	0.6 (-0.1-1.4)	0.103	0.9 (0.3-1.4)	0.2 (-0.6-1.4)	-2.6 (-4.8-0.0)
	≥70	-0.1 (-1.5-0.9)	-0.1 (-0.9-0.8)	0.499	0.5 (-0.4-0.8)	-1.3 (-2.6-0.2)	-0.6 (-1.0-0.0)
P for trend		**P < 0.001**	**P = 0.019**		P = 0.062	P = 0.424	P = 0.610
AL (mm)	30-39	23.2±0.8	21.8±0.0	0.072	-†	-†	-†
	40-49	23.2±0.9	22.0±0.4	0.001	22.0±0.4	-†	-†
	50-59	23.1±0.8	22.4±0.8	<0.001	22.5±0.8	21.4±1.0	22.3±1.0
	60-69	23.0±0.9	22.5±0.8	<0.001	22.5±0.9	22.5±0.7	22.1±0.6
	≥70	23.1±0.9	22.2±0.7	<0.001	22.2±0.6	21.9±1.1	22.7±0.6
P for trend		**P < 0.001**	P = 0.434		P = 0.306	P = 0.135	P = 0.681
ACD (mm)	30-39	3.1±0.4	2.4±0.0	0.090	-†	-†	-†
	40-49	2.9±0.4	2.3±0.2	<0.001	2.3±0.2	-†	-†
	50-59	2.8±0.4	2.4±0.3	<0.001	2.3±0.2	2.4±0.2	3.1±0.5
	60-69	2.6±0.4	2.3±0.3	<0.001	2.3±0.3	2.2±0.2	2.4±0.3
	≥70	2.6±0.4	2.3±0.3	<0.001	2.3±0.3	2.2±0.4	2.5±0.2
P for trend		**P < 0.001**	P = 0.548		P = 0.817	P = 0.544	P = 0.255
LT (mm)	30-39	4.4±0.6	4.6±0.0	0.658	-†	-†	-†
	40-49	4.6±0.3	4.9±0.3	0.016	4.9±0.3	-†	-†
	50-59	4.8±0.4	5.0±0.4	<0.001	5.0±0.3	4.8±0.4	4.4±0.5
	60-69	4.9±0.5	5.1±0.6	0.089	5.1±0.4	4.8±1.0	4.9±0.6
	≥70	5.0±0.6	5.2±0.3	0.127	5.3±0.3	5.2±0.4	5.0±0.2
P for trend		**P < 0.001**	P = 0.337		P = 0.101	P = 0.795	P = 0.650
Women							
SE (D)	30-39	-0.4 (-0.8-0.0)	-0.5 (-2.8-0.0)	0.438	-†	-0.4 (-0.5-0.0)	
	40-49	-0.3 (-0.5-0.1)	0.3 (-0.3-0.6)	0.615	0.3 (0.0-0.8)	-0.3 (-6.2-0.4)	-†
	50-59	0.1 (-0.4-0.5)	0.5 (0.1-1.0)	<0.001	0.5 (0.1-1.0)	0.5 (-0.3-1.3)	1.3 (1.3-1.3)
	60-69	0.6 (-0.3-1.3)	1.1 (0.6-1.8)	<0.001	1.1 (0.6-1.8)	1.3 (0.7-1.8)	0.1 (-0.5-0.0)
	≥70	0.4 (-1.1-1.6)	0.8 (-0.8-1.5)	0.040	0.9 (-0.2-1.5)	-0.6 (-1.8-1.2)	-2.9 (-4.4-0.0)
P for trend		**P < 0.001**	**P < 0.001**		**P = 0.004**	**P = 0.040**	P = 0.081
AL (mm)	30-39	22.7±0.8	22.1±0.4	0.192	-†	22.0±0.5	22.3±0.0
	40-49	22.6±0.7	22.2±1.6	0.011	21.9±0.7	23.3±3.3	-†
	50-59	22.6±0.8	22.1±0.7	<0.001	22.1±0.7	22.2±0.8	22.4±0.5
	60-69	22.6±0.9	22.0±0.7	<0.001	22.0±0.6	21.9±0.9	22.0±0.6
	≥70	22.7±1.0	22.2±0.6	<0.001	22.2±0.7	22.0±0.6	22.4±0.4
P for trend		**P = 0.048**	P = 0.491		P = 0.310	P = 0.220	P = 0.675
ACD (mm)	30-39	2.9±0.4	2.8±0.3	0.389	-†	2.9±0.2	2.5±0.0

	40-49	2.8±0.4	2.4±0.2	<0.001	2.4±0.2	2.5±0.1	-†
	50-59	2.6±0.4	2.3±0.3	<0.001	2.3±0.3	2.4±0.4	2.3±0.3
	60-69	2.5±0.4	2.2±0.3	<0.001	2.2±0.3	2.2±0.3	2.3±0.2
	≥70	2.5±0.4	2.1±0.3	<0.001	2.1±0.3	2.1±0.2	2.0±0.3
P for trend		**P < 0.001**	**P < 0.001**		**P = 0.001**	**P = 0.003**	P = 0.149
LT (mm)	30-39	4.3±0.5	4.5±0.4	0.631	-†	4.3±0.1	4.9±0.0
	40-49	4.5±0.4	4.8±0.3	0.001	4.8±0.4	4.8±0.2	-†
	50-59	4.7±0.4	4.9±0.4	<0.001	4.9±0.4	4.8±0.5	4.9±0.3
	60-69	4.9±0.5	5.0±0.4	0.001	5.1±0.3	5.0±0.4	4.9±0.5
	≥70	4.9±0.7	5.1±0.5	0.052	5.2±0.6	5.2±0.2	4.6±0.4
P for trend		**P < 0.001**	**P < 0.001**		**P < 0.001**	**P = 0.008**	P = 0.724
Both genders							
SE (D)	30-39	-0.4 (-0.8-0.0)	-0.9 (-2.4--0.4)	0.388	-†	-0.4 (-0.5-0.0)	-2.0 (-2.8-0.0)
	40-49	-0.3 (-0.5-0.1)	0.3 (-0.2-0.7)	0.801	0.3 (0.0-0.6)	-0.3 (-0.5-0.8)	-†
	50-59	0.1 (-0.4-0.5)	0.5 (0.1-1.0)	<0.001	0.5 (0.1-1.0)	0.5 (-0.2-1.3)	1.3 (-0.5-2.1)
	60-69	0.5 (-0.3-1.1)	1.0 (0.4-1.5)	<0.001	1.0 (0.5-1.6)	1.0 (0.3-1.5)	-0.5 (-2.6-0.9)
	≥70	0.0 (-1.3-1.1)	0.5 (-0.8-1.3)	0.007	0.8 (-0.3-1.4)	-0.6 (-2.0-0.9)	-1.2 (-3.6--0.4)
P for trend		**P < 0.001**	**P < 0.001**		**P = 0.008**	**P = 0.032**	P = 0.225
AL (mm)	30-39	22.9±0.8	22.1±0.3	0.033	-†	22.0±0.5	22.1±0.4
	40-49	22.9±0.9	22.2±1.5	<0.001	21.9±0.6	23.3±3.3	-†
	50-59	22.9±0.8	22.2±0.7	<0.001	22.2±0.7	22.1±0.9	22.4±0.6
	60-69	22.8±1.0	22.1±0.7	<0.001	22.1±0.7	22.1±0.9	22.0±0.6
	≥70	22.9±0.9	22.2±0.6	<0.001	22.2±0.6	22.0±0.7	22.5±0.5
P for trend		**P = 0.036**	P = 0.987		P = 0.396	P = 0.218	P = 0.398
ACD (mm)	30-39	3.0±0.4	2.7±0.3	0.092	-†	2.9±0.2	2.5±0.1
	40-49	2.8±0.4	2.4±0.2	<0.001	2.4±0.2	2.5±0.1	-†
	50-59	2.7±0.4	2.3±0.3	<0.001	2.3±0.3	2.4±0.3	2.6±0.5
	60-69	2.6±0.4	2.2±0.3	<0.001	2.2±0.3	2.2±0.3	2.3±0.2
	≥70	2.5±0.4	2.2±0.3	<0.001	2.2±0.3	2.1±0.3	2.1±0.4
P for trend		**P < 0.001**	**P < 0.001**		**P = 0.001**	**P = 0.001**	P = 0.118
LT (mm)	30-39	4.3±0.5	4.5±0.3	0.540	-†	4.3±0.1	4.8±0.2
	40-49	4.5±0.4	4.8±0.3	<0.001	4.9±0.4	4.8±0.2	-†
	50-59	4.8±0.4	4.9±0.4	<0.001	4.9±0.4	4.8±0.5	4.7±0.4
	60-69	4.9±0.5	5.1±0.4	<0.001	5.1±0.4	5.0±0.7	4.9±0.5
	≥70	5.0±0.7	5.1±0.5	0.022	5.2±0.5	5.2±0.2	4.7±0.4
P for trend		**P < 0.001**	**P < 0.001**		**P < 0.001**	P = 0.078	P = 0.795

PACD, primary angle closure disease; PACS, primary angle closure suspect; PAC, primary angle closure; PACG, primary angle closure glaucoma; OA, open angle; SE, spherical equivalent; AL, axial length; ACD, anterior chamber depth; LT, lens thickness. Only the right eye of persons with open angle, the only affected eye in those with unilateral glaucoma, and the more severely-affected eye in persons with bilateral glaucoma were included in analyses. *P-value was calculated by the t-test for normally distributed variables (i.e., AL, ACD, LT) and Mann-Whitney U test for non-

normally distributed variables (i.e., SE). † Not available due to limited sample size. Boldface values indicate statistical significance.

Supplementary Table S7. Comparison of biometric parameters between PACD patients with myopia and without myopia.

Variables	Age (years)	PACD without myopia	PACD with myopia	*P* value*
SE	30-39	0.1 (-0.3-0.5)	-1.3 (-2.1--0.6)	**0.003**
	40-49	0.3 (-0.1-0.5)	-0.8 (-1.0--0.6)	**<0.001**
	50-59	0.5 (0.1-1.0)	-0.6 (-0.9--0.5)	**<0.001**
	60-69	1.0 (0.5-1.6)	-1.0 (-2.1--0.5)	**<0.001**
	≥70	1.0 (0.4-1.9)	-1.6 (-2.8--1.0)	**<0.001**
	Total	0.8 (0.3-1.3)	-1.0 (-1.8--0.6)	**<0.001**
P for trend		**P < 0.001**	P = 0.067	
AL (mm)	30-39	22.4±0.5	21.8±0.8	0.248
	40-49	22.9±2.5	22.0±0.6	0.295
	50-59	22.4±0.9	22.3±0.7	0.539
	60-69	22.6±0.8	22.2±0.7	**0.005**
	≥70	22.4±0.7	22.2±0.7	0.052
	Total	22.5±1.1	22.2±0.7	**0.004**
P for trend		P = 0.611	P = 0.139	
ACD (mm)	30-39	2.6±0.2	2.6±0.3	0.742
	40-49	2.4±0.3	2.4±0.2	0.682
	50-59	2.4±0.3	2.3±0.3	0.335
	60-69	2.2±0.3	2.2±0.3	0.879
	≥70	2.2±0.3	2.3±0.3	0.054
	Total	2.3±0.3	2.3±0.3	0.678
P for trend		**P < 0.001**	P = 0.223	
LT (mm)	30-39	4.6±0.3	4.4±0.2	0.233
	40-49	4.9±0.2	4.7±0.3	0.112
	50-59	4.9±0.6	4.9±0.4	0.491
	60-69	4.9±0.9	5.1±0.4	0.221
	≥70	5.0±0.7	5.1±0.8	0.595
	Total	4.9±0.7	5.0±0.5	0.193
P for trend		P = 0.874	**P < 0.001**	

PACD, primary angle closure disease; SE, spherical equivalent; AL, axial length; ACD, anterior chamber depth; LT, lens thickness. Boldface values indicate statistical significance. *P-value was calculated by the t-test for normally distributed variables (i.e., AL, ACD, LT) and Mann-Whitney U test for non-normally distributed variables (i.e., SE).
